# On a New Class of *p*-Valent Meromorphic Functions Defined in Conic Domains

**DOI:** 10.1155/2016/6360250

**Published:** 2016-07-26

**Authors:** Mohammed Ali Alamri, Maslina Darus

**Affiliations:** School of Mathematical Sciences, Faculty of Science and Technology, Universiti Kebangsaan Malaysia, 43600 Bangi, Selangor, Malaysia

## Abstract

We define a new class of multivalent meromorphic functions using the generalised hypergeometric function. We derived this class related to conic domain. It is also shown that this new class of functions, under certain conditions, becomes a class of starlike functions. Some results on inclusion and closure properties are also derived.

## 1. Introduction

Let *M*_*p*_ denote the class of functions of the form

(1) $$\begin{array}{c} f\left(\;z\;\right)=\frac{1}{z^{p}}+\overset{\infty }{\underset{n=1}{\sum }}a_{n}z^{n-p},\;p\in N=1,2,3,\ldots , \end{array}$$

which are analytic and *p*-valent in the punctured unit disc centred at origin *E* ^*∗*^ = {*z* : 0 < |*z*| < 1} = *E*∖{0}. Also by *f*(*z*)≺*g*(*z*) we mean *f*(*z*) is subordinate to *g*(*z*) which implies the existence of an analytic function, called Schwartz function *w*(*z*) with |*w*(*z*)| < 1, for *z* ∈ *E* ^*∗*^ such that *f*(*z*) = *g*(*w*(*z*)), where *f*(*z*) and *g*(*z*) are multivalent meromorphic functions. Note that if *g* is univalent in *E* then the above subordination is equivalent to *f*(0) = *g*(0) and *f*(*E*) ⊂ *g*(*E*).

The set of points, for 0 < *γ* < 1 and *k* ∈ [0, *∞*,

(2) $$\begin{array}{c} \Omega_{k,\gamma }=\gamma \Omega_{k}+\left(\;1-\gamma \;\right), \end{array}$$

where

(3) $$\begin{array}{c} \Omega_{k}=\left\{\;u+iv:u>k\sqrt{{\left(u-1\right)}^{2}+v^{2}},\;u>0\;\right\}, \end{array}$$

[1] showed that the extremal functions *q*_*k*,*γ*_(*z*) for conic regions are convex univalent and given by

(4) $$\begin{array}{c} q_{k,\gamma }\left(\;z\;\right)=\left\{\begin{array}{cc} \frac{1+\left(1-2\gamma \right)z}{1-z}, & k=0,\\ 1+\frac{2\gamma }{1-k^{2}}\left[\frac{2}{\pi }\left(\arccos k\right)\mathrm{arctanh}\sqrt{z}\right], & 0<k<1,\\ 1+\frac{2\gamma }{\pi^{2}}{\left(\log\frac{1+\sqrt{z}}{1-\sqrt{z}}\right)}^{2}, & k=1,\\ 1+\frac{\gamma }{k^{2}-1}\left[\sin\left(\frac{\pi }{2R\left(t\right)}\overset{u\left(z\right)/\sqrt{t}}{\underset{0}{\int }}\frac{1}{\sqrt{1-x^{2}}\sqrt{1-{\left(tx\right)}^{2}}}dx\right)-1\right], & k>1, \end{array}\right. \end{array}$$

where *R*(*t*) is Legendre's complete elliptic integral of the first kind with $R^{\prime }(t)=\sqrt{1-t^{2}}$ as its complementary integral, $u(z)=(z-\sqrt{t})/(1-\sqrt{t}z)$, *t* ∈ (0,1), and *z* ∈ *E* is chosen in such a way that *k* = cosh⁡(*πR*′(*t*)/*R*(*t*)).

The generalised hypergeometric function _*q*_ *F*_*s*_(*α*_1_,…, *α*_*q*_; *β*_1_,…, *β*_*s*_; *z*) for complex parameters *α*_1_,…, *α*_*q*_ and *β*_1_,…, *β*_*s*_ with *β*_*j*_ ≠ 0, −1, −2, −3,…, for *j* = 1,2, 3,…, *s*, is defined as

(5) Fsqα1,…,αq;β1,…,βs;z=∑n=0∞α1n⋯αqnβ1n⋯βsnn!zn=1+∑n=1∞α1n⋯αqnβ1n⋯βsnznn!,

with *q* ≤ *s* + 1, *q*, *s* ∈ *ℕ*_0_ = *ℕ* ∪ {0}, and (*α*)_*n*_ is the well-known Pochhammer symbol related to the factorial and the Gamma function by the relation

(6) $$\begin{array}{c} {\left(\;\alpha \;\right)}_{n}=\frac{\left(\alpha +n-1\right)!}{\left(\alpha -1\right)!}=\frac{\Gamma \left(\alpha +n\right)}{\Gamma \left(\alpha \right)}. \end{array}$$

Also (5) implies

(7) hpα1,…,αq;β1,…,βs;z=z−pFsqα1,…,αq;β1,…,βs;z∈Mp.

Liu and Srivastava [2] defined a linear operator for functions belonging to the class of multivalent meromorphic function *H*_*p*_(*α*_1_,…, *α*_*q*_; *β*_1_,…, *β*_*s*_) : *M*_*p*_ → *M*_*p*_ as follows:

(8) Hpα1,…,αq;β1,…,βs=hpα1,…,αq;β1,…,βs;z⋆fz.

If we assume for brevity that *H*_*p*,*q*,*s*_(*α*_1_) = *H*_*p*_(*α*_1_,…, *α*_*q*_; *β*_1_,…, *β*_*s*_) then the following identity holds for this operator:

(9) zHp,q,sα1fz′=α1Hp,q,sα1+1fz−α1+pHp,q,sα1fz.

Shareef [3] defined and studied subclass *MQ*_*p*_(*k*, *λ*, *α*_1_) of meromorphic function associated with conic domain, for *k* ≥ 0, 0 ≤ *λ* < 1, and *p* ≥ 1, as follows:

(10) −1pzHp,q,sα1fz′+λz2Hp,q,sα1fz′′1−λHp,q,sα1fz+λzHp,q,sα1fz′≺qk,γz.

We now define a new subclass *MQ*_*p*_(*b*, *k*, *λ*, *α*_1_) of meromorphic function associated with conic domain, for *k* ≥ 0, 0 ≤ *λ* < 1, *p* ≥ 1, and *b* ≥ 1, as follows:

(11) −1p1+1bzHp,q,sα1fz′+λz2Hp,q,sα1fz′′1−λHp,q,sα1fz+λzHp,q,sα1fz′−b≺qk,γz.

Since *q*_*k*,*γ*_ is a convex and univalent function, for *h*(*z*)≺*q*_*k*,*γ*_(*z*) it means *h*(*E* ^*∗*^) is contained in *q*_*k*,*γ*_(*E* ^*∗*^), where

(12) hz=−1p1+1bzHp,q,sα1fz′+λz2Hp,q,sα1fz′′1−λHp,q,sα1fz+λzHp,q,sα1fz′−b.

In the next two sections, for brevity, we drop the subscripts of the operator *H*_*p*,*q*,*s*_(*α*_1_).

## 2. Preliminary Results

**Lemma 1 (see [4]).**

Let *h*_2_(*z*) be convex in *E* and *ℜ*(*λh*_2_(*z*) + *μ*) > 0, where *μ* ∈ *ℂ*, *λ* ∈ *ℂ*∖{0}, and *z* ∈ *E*. If *h*_1_(*z*) is analytic in *E*, with *h*_1_(0) = *h*_2_(0), then

(13) h1z+zh1′zλh1z+μ≺h2zimplies  h1z≺h2z.

**Lemma 2 (see [5]).**

Let *h*(*z*) = 1 + ∑_*n*=1_^*∞*^ *c*_*n*_ *z* ^*n*^ and *H*(*z*) = 1 + ∑_*n*=1_^*∞*^ *d*_*n*_ *z* ^*n*^ and *h*≺*H*. If *H*(*z*) is univalent and convex in *E*, then

(14) $$\begin{array}{c} \left|\;c_{n}\;\right|\leq \left|\;d_{1}\;\right|, \end{array}$$

for *n* ≥ 1.

**Lemma 3 (see [6]).**

If *q*_*k*,*γ*_(*z*) = 1 + *q*_1_ *z* + *q*_2_ *z* ^2^ + ⋯ then

(15) $$\begin{array}{c} q_{1}=\left\{\begin{array}{cc} \frac{8\left(1-\gamma \right){\left(\arccos\left(k\right)\right)}^{2}}{\pi^{2}\left(1-k^{2}\right)}, & 0<k<1,\\ \frac{8\left(1-\gamma \right)}{\pi^{2}}, & k=1,\\ \frac{\pi^{2}\left(1-\gamma \right)}{4\sqrt{t}\left(k^{2}-1\right)k^{2}\left(t\right)\left(1+t\right)}, & k>1. \end{array}\right. \end{array}$$

One now states and proves the main results.

## 3. Main Results

In this section we explore some of the geometric properties exhibited by the class *MQ*_*p*_(*b*, *k*, *λ*, *α*_1_).

We begin by discussing an inclusion property for the class *MQ*_*p*_(*b*, *k*, *λ*, *α*_1_).

**Theorem 4 .**

If *ℜ*(*α*_1_) > *pℜ*(*bq*_*k*,*λ*_(*z*) − 1) then

(16) $$\begin{array}{c} MQ_{p}\left(\;b,k,\lambda ,\alpha_{1}+1\;\right)\subset MQ_{p}\left(\;b,k,\lambda ,\alpha_{1}\;\right). \end{array}$$

**Proof**

Let *f* ∈ *MQ*_*p*_(*b*, *k*, *λ*, *α*_1_ + 1) and set

(17) −1p1+1bzHα1fz′+λz2Hα1fz′′1−λHα1fz+λzHα1fz′−b=hz.

But differentiating (9) with respect to *z* we get

(18) zHα1fz′=α1Hα1+1fz′−α1+pHα1fz′,zHα1fz′′+Hα1fz′=α1Hα1+1fz′−α1+pHα1fz′,z2Hα1fz′′=α1zHα1+1fz′−α1+p+1zHα1fz′.

Putting (18) in (17) we have

(19) λzα1Hα+1fz′+1−λα1+p+1zHα1fz′1−λHα1fz+λzHα1fz′=−bphz,λα1zHα1+1fz′+1−λα1Hα1+1fz1−λHα1fz+λzHα1fz′=−bphz+α1+p.

Taking logarithmic derivative of (19) we have

(20) zHα1+1fz′+λz2Hα1+1fz′′1−λHα1+1fz+λzHα1+1fz′+bphz+bb−1=−bpzh′z−bphz+α1+p,zHα1+1fz′+λz2Hα1+1fz′′1−λHα1+1fz+λzHα1+1fz′=−bphz+zh′z−bphz+α1+p−bb−1,−1p1+1bzHα1+1fz′+λz2Hα1+1fz′′1−λHα1+1fz+λzHα1+1fz′−b=hz+zh′z−bphz+α1+p.

Since *f* ∈ *MQ*_*q*_(*b*, *k*, *λ*, *α*_1_ + 1), therefore

(21) $$\begin{array}{c} h\left(\;z\;\right)+\frac{zh^{\prime }\left(z\right)}{-bph\left(z\right)+\left(\alpha_{1}+p\right)}≺q_{k,\gamma }\left(\;z\;\right). \end{array}$$

Using Lemma 2 we have

(22) $$\begin{array}{c} h\left(\;z\;\right)≺q_{k,\gamma }\left(\;z\;\right), \end{array}$$

provided *ℜ*(−*bpq*_*k*,*λ*_(*z*) + *α*_1_ + *p*) > 0 or equivalently *ℜ*(*α*_1_) > *pℜ*(*bq*_*k*,*γ*_(*z*) − 1). Hence, *f* ∈ *MQ*_*p*_(*b*, *k*, *λ*, *α*_1_).

We now show that the class *MQ*_*p*_(*b*, *k*, *λ*, *α*_1_) is closed under a certain integral.

**Theorem 5 .**

If *f*(*z*) ∈ *MQ*_*p*_(*b*, *k*, *λ*, *α*_1_), then the integral

(23) $$\begin{array}{c} B_{\eta }\left(\;H\left(\;\alpha \;\right)f\left(\;z\;\right)\;\right)=\frac{\eta -p}{z^{\eta }}\overset{z}{\underset{0}{\int }}t^{\eta -1}\left(\;H\left(\;\alpha \;\right)f\left(\;z\;\right)\;\right)dt \end{array}$$

maps *f*(*z*) into *MQ*_*p*_(*b*, *k*, *λ*, *α*_1_).

**Proof**

From (23) we have

(24) $$\begin{array}{c} z^{\eta }B_{\eta }\left(\;H\left(\;\alpha \;\right)f\left(\;z\;\right)\;\right)=\left(\;\eta -p\;\right)\overset{z}{\underset{0}{\int }}t^{\eta -1}\left(\;H\left(\;\alpha \;\right)f\left(\;z\;\right)\;\right)dt. \end{array}$$

Note that

(25) $$\begin{array}{c} B_{\eta }\left(\;H\left(\;\alpha \;\right)f\left(\;z\;\right)\;\right)=\left(\;H\left(\;\alpha \;\right)\;\right)B_{\eta }f\left(\;z\;\right). \end{array}$$

Differentiating (24) above we get

(26) ηzη−1BηHαfz+zBηHαfz′=η−pzη−1Hαfz,ηBηHαfz+zBηHαfz′=η−pHαfz.

Differentiate again

(27) ηBηHαfz′+zBηHαfz′′+BηHαfz′=η−pHαfz′,zBηfz′′=η−pBηHαfz′−η+1Bηfz′.

Now let

(28) −1p1+1bzBηHα1f′+λz2BηHα1f′′1−λBηHα1f+λzBηHα1f′−b=gz.

Using (26) and (27) in (28) we get

(29) zBηHα1f′+λzη−pHα1f′−z1+ηBηHα1f′1−λBηHα1f+λzBηHα1f′=−bpgz+b2−b,λzη−pHα1f′+1−λ1+ηη−pHα1f−ηBηHα1f1−λBηHα1f+λzBηHα1f′=−bpgz+b2−b,η−pλzHα1f′−1−λHα1f−ληzBηHα1f′−1−ληBηHα1f1−λBηHα1f+λzBηHα1f′=−bpgz+b2−b,η−pλzHα1f′−1−λHα1f1−λBηHα1f+λzBηHα1f′=−bpgz+b2−b+η.

Now taking logarithmic derivative we have

(30) η−pλzHα1f′′+λHα1f′+1−λHα1f′η−pλzHα1f′−1−λHα1f−1−λBηHα1f′+λzBηHα1f′′+λBηHα1f′1−λBηHα1f+λzBηHα1f′=−bpg′z−bpgz+b2−b,−1p1+1bzHα1f′+λz2Hα1f′′1−λHα1f+λzHα1f′−b=gz+zg′z−bpgz+b2−b.

Using Lemma 2 we get *g*(*z*)≺*q*_*k*,*γ*_(*z*) which implies

(31) $$\begin{array}{c} B_{\eta }\left(\;f\left(\;z\;\right)\;\right)\in MQ_{p}\left(\;b,k,\lambda ,\alpha_{1}\;\right). \end{array}$$

This proves the assertion.

Now we get coefficient estimates of the class *MQ*_*p*_(*b*, *k*, *λ*, *α*_1_).

**Theorem 6 .**

If *f*(*z*) ∈ *MQ*_*p*_(*b*, *k*, *λ*, *α*_1_) and *f*(*z*) is given by (1) then

(32) $$\begin{array}{c} \left|\;a_{n}\;\right|\leq \left|\;\frac{bpq_{1}}{b\left(1-b\right)+bp-p+n}\;\right|\overset{n-1}{\underset{k=0}{\sum }}\left|\;a_{k}\;\right|,\;a_{0}=1 \end{array}$$

provided

(33) 2λp−1+3−nλ<kλfor  1−λp+n−1λ>0,2λp−1+3−nλ>kλfor  1−λp+n−1λ<0,

for all *k* ≤ *n*.

**Proof**

Let *f*(*z*) ∈ *MQ*_*p*_(*b*, *k*, *λ*, *α*_1_); then, by definition, we have

(34) −1p1+1bzHα1fz′+λz2Hα1fz′′1−λHα1fz+λzHα1fz′−b=hz≺qk,γz,−zHα1fz′+λz2Hα1fz′′p1−λHα1fz+λzHα1fz′=bhz+bp−b2p,

which gives

(35) −zHα1fz′+λz2Hα1fz′′p=bhz+b1−bp·1−λHα1fz+λzHα1fz′.

Let us write *H*(*α*_1_)*f*(*z*) = *f*(*z*); then

(36) fz=z−p+∑n=0∞anzn−p,f′z=−pz−p−1+∑n=0∞ann−pzn−p−1,zf′z=−pz−p+∑n=0∞ann−pzn−p,f′′z=−p−p−1z−p−2+∑n=0∞ann−pn−p−1zn−p−2,z2f′′z=−p−p−1z−p+∑n=0∞ann−pn−p−1zn−p.

Assuming *h*(*z*) = 1 + ∑_*n*=1_^*∞*^ *c*_*n*_ *z* ^*n*^, then (35) becomes

(37) z−p−∑n=0∞ann−ppzn−p+λ−p+1z−p+∑n=0∞ann−pn−p−1pzn−p,1−λp−λz−p−∑n=1∞ann−pp1+λn−p−1zn−p=b+∑n=1∞bcnzn+b1−bp1−λp−λz−p+∑n=1∞an1+λn−p−1zn−p.

From (37) we have

(38) 1−λp−λz−p−∑n=1∞ann−pp1+λn−p−1zn−p=b+bc1z+bc2z2+bc3z3+⋯+bcnzn+⋯+b1−bp·1−λp−λz−p+∑n=1∞an1+λn−p−1zn−p.

Now comparing coefficients of *z* ^1−*p*^ we have

(39) −a11−pp1−λp=a1b1−λp+a1b1−bp·1−λp+bc11−λp−λ,−a11−λpb1−b+bp−p+1p=bc11−λp−λ,a1=−p1−λpb1−b+bp−p+11−λp−λ·bc1,

and comparing the coefficients of *z* ^2−*p*^ gives

(40) −a22−pp1+λ1−p=a21+λ1−pb+b1−bp+a11−λpbc1+bc21−λp−λ,−a21+λ1−p2−pp+bp+b1−bp=a11−λpbc1+bc21−λp−λ,−a21+λ1−pb1−b+bp−p+2p=a11−λpbc1+bc21−λp−λ,a2=−p1+λ1−pb1−b+bp−p+21−λp−λbc2+1−λpba1c1,

and for the coefficient of *z* ^3−*p*^ we have

(41) −a31+λ2−pb1−b+bp−p+3p=bc1a21+λ1−p+bc2a11−λp+bc31−λp−λ,a3=−p1+λ2−pb1−b+bp−p+31−λp−λbc3+1−λpbc2a1+1+λ1−p·bc1a2,

which generalise to

(42) an=−p1−λp+n−1λb1−b+bp−p+n1−λp−λbcn+1−λpbcn−1a1+1−λp+λ·bcn−2a2+⋯+1−λp+n−2λbc1an−1.

The above expression can also be written as

(43) an−p1−λp+n−1λb1−b+bp−p+n·∑k=1n1−λp+k−2λcn−k−1ak−1,with  a0=1.

Now taking

(44) $$\begin{array}{c} A_{n,k}=-\frac{1-\lambda p+\left(k-2\right)\lambda }{1-\lambda p+\left(n-1\right)\lambda },\;n\geq k, \end{array}$$

we have

(45) An,k<1,if  2λp−1+3−nλ<kλfor  1−λp+n−1λ>0,2λp−1+3−nλ>kλfor  1−λp+n−1λ<0,

for all *n* ≥ *k*. Since *q*_*k*,*γ*_(*z*) is univalent and *q*_*k*,*γ*_(*E*) is convex, applying Rogosinski's theorem we have

(46) $$\begin{array}{c} \left|\;c_{n}\;\right|\leq \left|\;q_{1}\;\right|, \end{array}$$

where *q*_1_ is given in (15). Under the conditions given in (45), expressions (39)–(42) give

(47) a1≤bpq1b1−b+bp−p+1,a2≤bpq1b1−b+bp−p+21+a1,a3≤bpq1b1−b+bp−p+31+a1+a2,⋮an≤bpq1b1−b+bp−p+n·1+a1+a2+⋯+an−1.

This can also be written as

(48) $$\begin{array}{c} \left|\;a_{n}\;\right|\leq \left|\;\frac{bpq_{1}}{b\left(1-b\right)+bp-p+n}\;\right|\overset{n-1}{\underset{k=0}{\sum }}\left|\;a_{k}\;\right|. \end{array}$$

This concludes the proof.
